# Novel and Diverse Non-Rabies Rhabdoviruses Identified in Bats with Human Exposure, South Dakota, USA

**DOI:** 10.3390/v12121408

**Published:** 2020-12-08

**Authors:** Ben M. Hause, Eric Nelson, Jane Christopher-Hennings

**Affiliations:** Animal Disease Research and Diagnostic Laboratory, Department of Veterinary and Biomedical Sciences, South Dakota State University, Brookings, SD 57007, USA; eric.nelson@sdstate.edu (E.N.); jane.hennings@sdstate.edu (J.C.-H.)

**Keywords:** rhabdovirus, bat, vesiculovirus, rabies

## Abstract

Bats are a host and reservoir for a large number of viruses, many of which are zoonotic. In North America, the big brown bat (*Eptesicus fuscus*) is widely distributed and common. Big brown bats are a known reservoir for rabies virus, which, combined with their propensity to roost in human structures, necessitates testing for rabies virus following human exposure. The current pandemic caused by severe acute respiratory syndrome coronavirus 2, likely of bat origin, illustrates the need for continued surveillance of wildlife and bats for potentially emerging zoonotic viruses. Viral metagenomic sequencing was performed on 39 big brown bats and one hoary bat submitted for rabies testing due to human exposure in South Dakota. A new genotype of American bat vesiculovirus was identified in seven of 17 (41%) heart and lung homogenates at high levels in addition to two of 23 viscera pools. A second rhabdovirus, Sodak rhabdovirus 1 (SDRV1), was identified in four of 23 (17%) viscera pools. Phylogenetic analysis placed SDRV1 in the genus *Alphanemrhavirus*, which includes two recognized species that were identified in nematodes. Finally, a highly divergent rhabdovirus, Sodak rhabdovirus 2 (SDRV2), was identified in two of 23 (8.7%) big brown bats. Phylogenetic analysis placed SDRV2 as ancestral to the dimarhabdovirus supergroup and *Lyssavirus*. Intracranial inoculation of mouse pups with rhabdovirus-positive tissue homogenates failed to elicit clinical disease. Further research is needed to determine the zoonotic potential of these non-rabies rhabdoviruses.

## 1. Introduction

Long recognized as a reservoir of rabies virus, bats are the host to an ever-expanding cadre of zoonotic viruses [1]. Repeated spillover of coronaviruses, paramyxoviruses, and filoviruses from bats to humans have been documented, likely including the current worldwide pandemic caused by severe acute respiratory syndrome coronavirus 2 [2,3]. Multiple factors contribute to the diversity of viruses in bats, including their ancient origin, species diversity and abundance, ability to fly, and inhabitation of diverse ecosystems [4].

The family *Rhabdoviridae* is comprised of 20 genera and 144 recognized species identified from a wide range of hosts including plants, insects, amphibians, fish, and mammals [5]. A number of species in the genus *Lyssavirus*, which includes rabies virus, exist in bat or wildlife reservoirs and are zoonotic, causing encephalitis. Members of the genera *Ledantevirus* (Le Dantec virus) and *Vesiculovirus* (vesicular stomatitis viruses, Isfahan virus, Chandipura virus), and possibly *Tibrovirus*, are largely arboviruses that can variably infect humans and cause febrile illness or encephalitis [6,7].

Members of multiple genera of *Rhabdoviridae* are found in bats. The most studied genus, *Lyssavirus*, includes numerous species with a bat reservoir that can infect a wide array of vertebrates [8]. Besides *Lyssavirus*, several members of *Ledantevirus* have been identified in bats, including Le Dantec virus, Kern Canyon virus, Mount Elgon bat virus, Vaprio virus, and Kumasi rhabdovirus [7,9,10,11]. Next-generation sequencing has also identified bat *Vesiculovirus* species, as well as divergent strains phylogenetically distinct from established genera [12,13].

## 2. Materials and Methods

### 2.1. Bat Collection and Sample Processing

**Figure 1:**
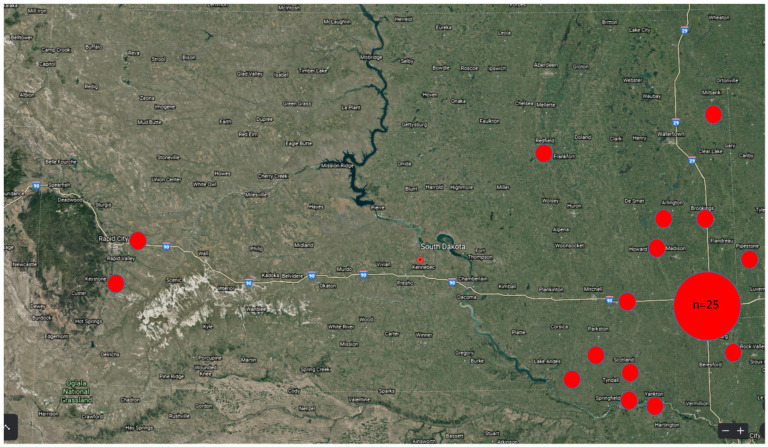
Map of bat collection sites. Each small red dot represents a single bat specimen. The large red dot represents 25 specimens. Map of South Dakota downloaded from Google Earth (www.google.com/earth).

To identify novel, possibly zoonotic viruses in bats with human exposure, metagenomic sequencing was performed on 39 big brown bats (*Eptesicus fuscus*) and one hoary bat (*Aeorestes cinereus*) submitted to the South Dakota State University Animal Disease Research and Diagnostic Laboratory (SDSU ADRDL) for rabies testing due to human exposure between March and June 2020. Thirty-nine of the bats were collected in South Dakota, and one was collected in Minnesota (Figure 1). For 17 of 40 bats, a tissue homogenate pool was assembled from heart and lungs. For the remaining bats (*n* = 23), a tissue homogenate was prepared from all viscera.

### 2.2. Viral Metagenomic Sequencing

Clarified homogenates were treated with nucleases followed by nucleic acid isolation. Reverse transcription and second strand synthesis were performed with barcoded random hexamers followed by amplification with barcode primers. Sequencing libraries were constructed with a Nextera XT library preparation kit followed by sequencing with a MiSeq instrument. Individual sequencing libraries were prepared for each sample prior to pooling of 10 to 25 samples for a MiSeq run. Bat sequencing libraries were pooled with non-bat specimens included in the same run. Bat specimens were sequenced on multiple MiSeq runs. Approximately 0.21–1.0 million paired 151 base pair (bp) reads were generated per sample. Contigs were assembled de novo using CLC Genomics and analyzed by BLASTX using the BLAST2Go plugin and the non-redundant protein sequence database in May 2020.

### 2.3. Phylogenetic Analysis

Phylogenetic analysis was performed on conserved regions (Gblocks) of the L protein from 135 members of *Rhabdoviridae* [5,14,15]. Sequences (435 amino acids) were aligned by MUSCLE, and the phylogeny was inferred with the maximum likelihood method using the Whelan and Goldman (WAG) + frequency model incorporated into MEGAX, with the tree topology assessed with 500 bootstrap replicates.

### 2.4. RT-PCR Detection of Viruses

To assess the prevalence of American bat vesiculovirus (ABVV) in *E. fuscus*, a Taqman assay was designed to target the conserved L gene (ABVV: forw 5′-ACACCCCTTCAAATCTTCCTC-3′; rev 5′-TGACACTCAATGACACCTCTG; probe 5′-FAM-CACACTCCCGCTGTATTCTCGCC). Similarly, Taqman assays were designed to detect Sodak rhabdovirus 2 (forw 5′-TCACTTGTCGGAAATCCTGG; rev 5′-TTCTGTTATGCTGGCTCTCAG; probe 5′-FAM-AAGTCACATTCCTTGGGTCGAGCTG-3′) and Sodak rhabdovirus 1 (forw 5′-GGCCTAAAACGCTCATTTCAC-3′; rev 5′-AGACCCCAACAGTTCTAAAGG; probe 5′-FAM-TGTTAAATCCCCATGAAGCTGCCCT-3′). Real-time RT-PCR was performed using the Taqman FAST virus 1-step master mix following the manufacturer’s suggested protocol.

### 2.5. Virus Isolation

Virus isolation was attempted for samples positive for ABVV, SDRV1, and SDRV2 on Vero and immortalized *Eptesicus fuscus* kidney (Efk3) cells [16]. Cells were propagated in DMEM media supplemented with 10% fetal bovine serum and grown at 37 °C with 5% CO_2_. Confluent monolayers in 12-well plates were inoculated with 100 µL of 0.22 µm filtered tissue homogenate and incubated for seven days before passaging to new cells. Cell culture supernatants were passaged one time to a new monolayer of cells and incubated for seven days before being considered negative when no cytopathic effects were apparent.

### 2.6. Mouse Inoculation Test

Pregnant CD-1 mice were purchased from Charles River Laboratories. A single female was caged with her litter. Following parturition, three-day-old suckling mice were injected intracranially with 10 µL of 0.22 µm sterile filtered bat tissue homogenate [17]. A single homogenate was used to inoculate an entire litter of pups. Pups were observed three times daily for morbidity and mortality for 18 days.

### 2.7. Ethics Statement

Mouse studies were approved by the South Dakota State University Institutional Animal Use and Care Committee (Approval Number 2008-036A, 25 August 2020). Bat specimens were submitted to the SDSU ADRDL for diagnostic testing, and as such, no specific approval was necessary.

## 3. Results

### 3.1. American Bat Vesiculovirus Is Common in Heart and Lung Tissue Homogenates

An approximately 10.7 kbp contig was assembled de novo each in four heart and lung tissue homogenates from big brown bats submitted for rabies testing (Table S1). BLASTX analysis identified ~90% identity to the RNA-dependent RNA polymerase of American bat vesiculovirus (ABVV). BLASTn analysis of the contigs found 77% identity to ABVV. These ABVV genome sequences were submitted to GenBank under Accession Numbers MT561344-MT561347. The highly conserved nucleocapsid (N) protein was 97% identical to the sole representative of ABVV, strain TFFN-2013, similarly identified in big brown bats [12]. Greater sequence divergence was observed for the ABVV glycoprotein (G), with only 74% identity to strain TFFN-2013. BLASTP analysis of the remaining phosphoprotein (P), matrix (M), and polymerase (L) proteins found 80%, 88%, and 91% identity, respectively, to TFFN-2013 (Table S2). The four ABVV genome sequences were greater than 99% identical to one another (Table S3).

### 3.2. Novel Rhabdoviruses Identified in Bat Viscera Homogenates

Sequence analysis of a viscera pool identified a 11,221 bp contig that was 46–54% identical to nematode rhabdovirus genomes spanning the L protein (Table S1). Open reading frame (ORF) and BLASTP analysis identified five putative proteins that were all most similar to Xingshan nematode virus 4 N, P, M, G, and L proteins, with percent identities of 47.6%, 25.3%, 29.4%, 24.8%, and 53.6%, respectively (Table S2). Genes encoding the predicted N and P proteins overlapped by 62 nucleotides. Given its genetic similarity to rhabdoviruses, the virus was named Sodak rhabdovirus 1 (SDRV1) and submitted to GenBank under Accession MT875151.

Analysis of a second viscera pool found a 12,600 bp contig that had ~33–45% identity to rhabdovirus L proteins over the 3′-half of the contig (Table S1). BLASTP analysis of predicted open reading frames identified the canonical rhabdovirus genome architecture with N, G, and L proteins displaying low (~24–42%) homology to rhabdoviruses (Table S2). The virus was named Sodak rhabdovirus 2 (SDRV2) and submitted to GenBank under Accession MT732687. Putative ORFs for P and M, identified based on their location in the genome, showed no similarity to known proteins. A 75 amino acid (aa) ORF, by convention designated unknown 1 (U1), was identified between the G and L genes and likewise had no similarity to known proteins. The related *Almendravirus* encodes a class 1a viroporin of a similar size between the G and L genes; it is unknown if a similar function is encoded by SDRV2 U1 [5]. The six genes (3′-N, P, M, G, U1, L-5′) were each part of separate transcriptional units, with conserved transcriptional initiation and termination/polyadenylation sites [18].

### 3.3. Phylogenetic Analysis

**Figure 2:**
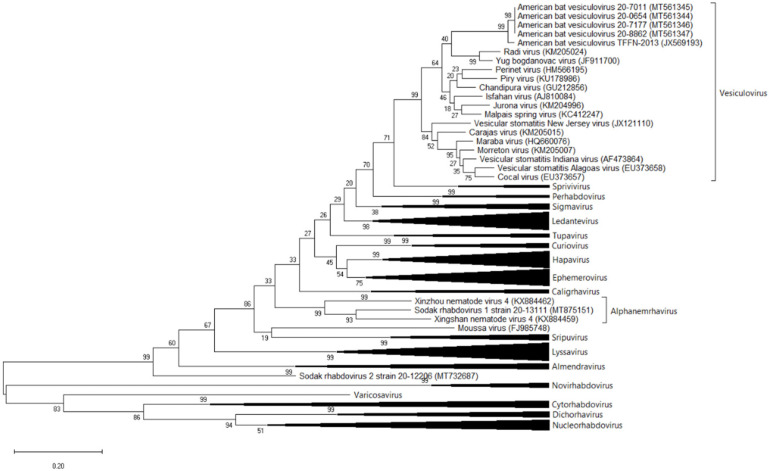
Phylogenetic analysis of conserved blocks of the L protein of *Rhabdoviridae*. Sequences were aligned by MUSCLE, and the phylogenetic tree was inferred by maximum likelihood analysis using the Whelan and Goldman model using a single set of stationary frequencies as implemented in MEGAX. Tree topology was verified with 500 bootstrap replicates.

The four strains of ABVV were closely related to the single previously identified strain of ABVV that together formed a sister clade to one comprised of Radi vesiculovirus and Yug Bogdanovac vesiculovirus, both identified from sandflies (Figure 2). Together, these viruses were related to the human pathogenic vesiculoviruses Chandipura and Isfahan viruses. Sodak rhabdovirus 1 clustered with Xinzhou nematode virus 4 and Xingshan nematode virus 4, the only two recognized species in the genus *Alphanemrhavirus*. Sodak rhabdovirus 2 formed a distinct lineage in a basal position to *Almendravirus*, *Lyssavirus* and the dimarhabdovirus (rhabdoviruses that replicate in dipteran and mammalian species) supergroup.

### 3.4. Molecular Detection of Bat Rhabdoviruses

Seven out of 17 (41%) heart and lung pools were positive for ABVV with *C*t values 11–33, indicating that ABVV infection of bats in South Dakota is common and includes a high titer viremia. Only three out of 23 (13%) viscera pools were positive for ABVV.

Sodak rhabdovirus 1 was only detected in four of the 23 viscera pools (17%). Similarly, SDRV2 was only identified in two of the 23 viscera pools (8.7%). Cycle threshold values for SDRV1 were 24–32 and were 25–34 for SDRV2.

### 3.5. Mouse Inoculation

Intracranial inoculation of mouse pups with ABVV, SDRV1, and SDRV2 was conducted to evaluate potential virus pathogenicity. A complete litter comprised of 13, 11, or 12 pups was inoculated with each virus, respectively, and observed for 18 days. No clinical signs of disease or mortality were observed.

### 3.6. Virus Isolation

Rhabdovirus-positive tissue homogenates were used to inoculate Vero and Efk3 cells. No cytopathic effects (CPE) were evident following seven days of incubation. Likewise, no CPE was evident after seven days following passage of cell culture supernatants to a new monolayer of Vero and Efk3 cells.

## 4. Discussion

Rhabdoviruses from multiple genera cause disease in humans, including rabies virus, which is responsible for an excess of 50,000 human fatalities annually [19]. Bats are a reservoir for rabies virus and the most commonly detected rabies virus carrier detected following human exposure testing. Here, we utilized bats submitted for rabies testing to identify other potential zoonotic viruses.

American bat vesiculovirus, previously identified in *E. fuscus*, was found in 23% of bats [12]. While the P, M, N, and L proteins displayed 80% or greater identity to the only strain of ABVV previously identified TFFN-2013, the surface glycoprotein G was only 74% identical, suggesting that significant genetic diversity exists within the species. Interestingly, ABVV was identified in 41% of heart and lung homogenates with cycle threshold values as low at 11. A lower frequency of detection (13%) and higher *C*t values were found in viscera pools. These results suggest that ABVV infects bats as opposed to detection due to environmental or dietary contamination. While the zoonotic potential of ABVV is unknown, several members of *Vesiculovirus* cause disease in humans. Chandipura virus, for example, has caused outbreaks of encephalitis in children in India [20,21]. Another outstanding question is the route of bat infection with ABVV. Most members of *Vesiculovirus* are arboviruses, suggesting a possible insect reservoir for ABVV. The high titer of ABVV in bat cardiac and pulmonary tissue also raises questions on possible adverse health effects on bats.

*Alphanemrhavirus* is comprised of two species, Xingshan alphanemrhavirus and Xinzhou alphanemrhavirus, both identified by metagenomic sequencing of nematodes [22]. Nematodes are known to parasitize bats [23], including bats in the genus *Eptesicus* [24,25]. Nematodes have been identified in both bat abdominal and thoracic cavities. A survey of helminths parasitizing three bat species roosting in a cave in Mexico found that nematodes comprised 20.7% of the helminths identified [26]. Sodak rhabdovirus 1 occupied a phylogenetically intermediate position between Xingshan and Xinzhou alphanemrhaviruses, suggesting a likely nematode origin. The sample in which Sodak rhabdovirus 1 was identified consisted of pooled viscera that was homogenized following dissection. No attempts were made to identify endoparasites, and the intestinal contents were not separately observed. Nematode body sizes can vary from microscopic to meters in length; however, many nematodes parasitizing bats have been ~9–28 mm in length [25]. Further research is needed to determine whether SDRV1 is capable of infecting bats or rather infects nematodes parasitizing bats. Interestingly, members of *Ledantevirus* were identified both in Nycteribiid bat flies and in the Angolan soft-furred fruit bats that they parasitize [27].

Phylogenetic analysis suggests that SDRV2 is derived from an ancestral virus precursor of *Almendravirus*, *Lyssavirus*, and the dimarhabdovirus supergroup. Almendraviruses are only known to infect mosquitoes, while lyssaviruses exist in a bat reservoir and infect other mammals [28]. Dimarhabdoviruses possess a wide array of lifecycles with hosts ranging from vertebrates to invertebrates; many are arboviruses. The finding of 8.7% positive bat viscera pools, and no heart and lungs pools, raises questions as to whether SDRV2 infects bats or rather was acquired through insects by feeding. Further research is needed to ascertain the host of SDRV2 and its potential for zoonosis.

Virus isolation using immortalized cell lines and suckling mice were unsuccessful. Suckling mice have long been used for rhabdovirus research, as they are highly susceptible to virus infection that results in high viral titers and clinical disease. While ABVV, SDRV1, and SDRV2 all failed to cause disease here, further studies, including serosurveys, are needed to evaluate the potential host range of these viruses.

A viral metagenomic survey in China likewise reported diverse rhabdoviruses in bats [13]. Two vesiculoviruses, Jinghong bat virus IH17 and Benxi bat virus GH1, most similar to ABVV, were identified in *Rhinolophidae* bats. These viruses met the criteria for representing a distinct species. Additionally, a highly divergent virus, Yangjiang bat virus (YJBV), was identified in multiple *Hipposideridae* bats. While only a partial L gene sequence was determined, phylogenetic analysis placed YJBV in a sister clade relationship to dimarhabdoviruses similar to SDRV2. Detection of novel vesiculoviruses and divergent unclassified rhabdoviruses in multiple bat genera in different countries suggests bats may constitute an under-appreciated reservoir for diverse *Rhabdoviridae*.

## Supplementary Materials

The following are available online at https://www.mdpi.com/1999-4915/12/12/1408/s1, Figure S1: Sequencing reads mapping to *Rhabdovirus* genomes assembled de novo, Table S1: Next generation sequencing metrics for sampling containing rhabdoviruses, Table S2: BLASTP analysis of putative open reading frames, Table S3: Nucleotide pairwise identity for ABVV strain genome sequences.
