# Supplementary material for: Novel and Diverse Non-Rabies Rhabdoviruses Identified in Bats with Human Exposure, South Dakota, USA
Source: Viruses. 2020 Dec 8;12(12):1408. doi: 10.3390/v12121408 (PMC7762532; doi:10.3390/v12121408)
Supplement: Supplementary file 1 [file viruses-12-01408-s001.pdf]

viruses

Supplementary Materials:

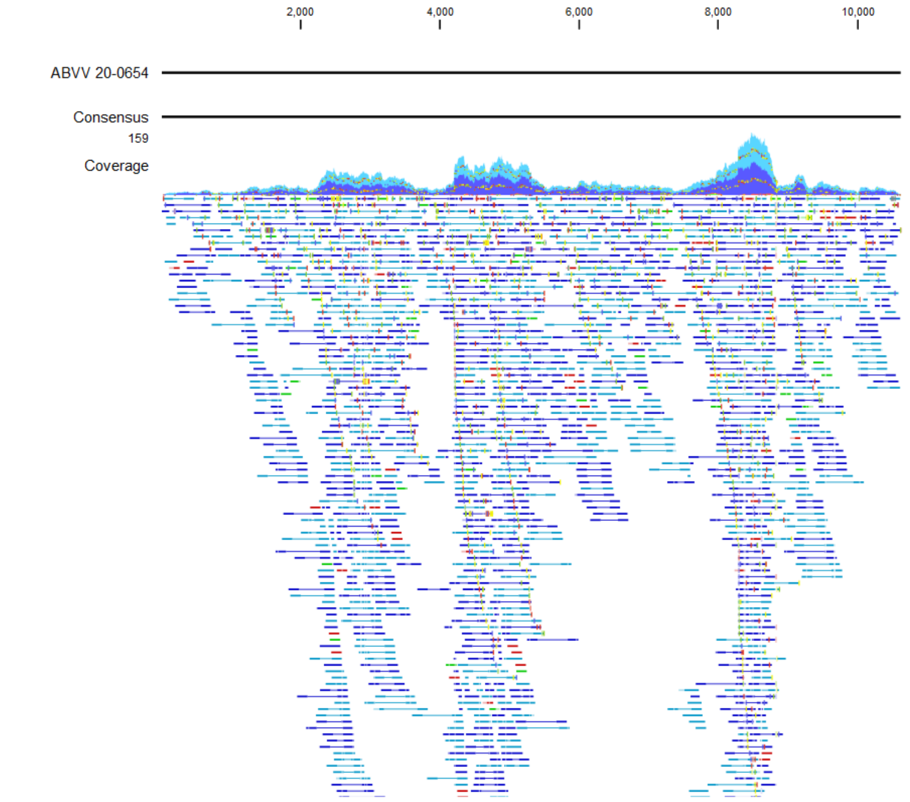

a

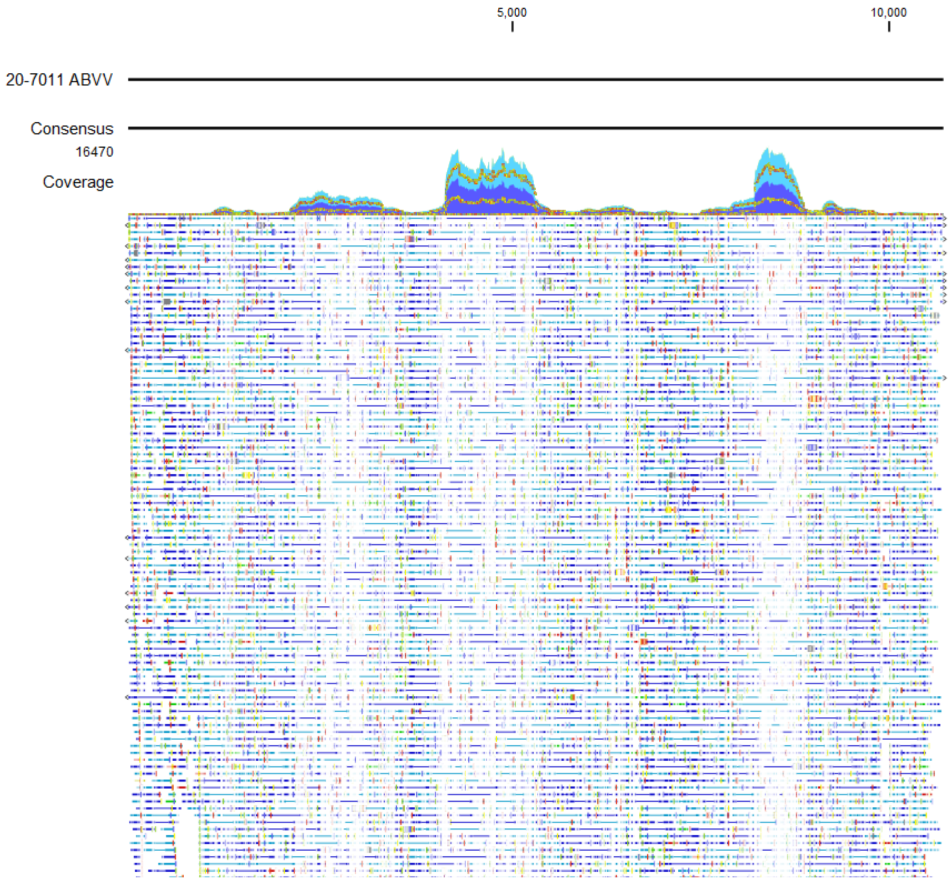

b

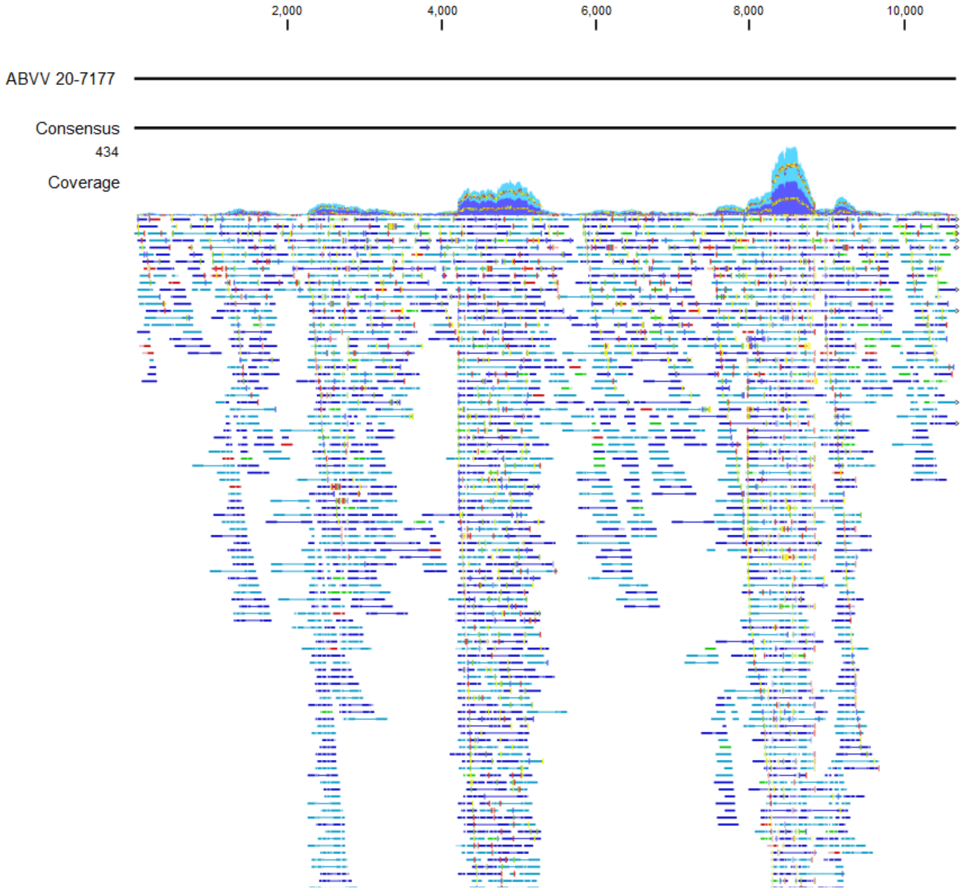
2,000
4,000
6,000
8,000
10,000
ABVV 20-7177
Consensus
434
Coverage

c

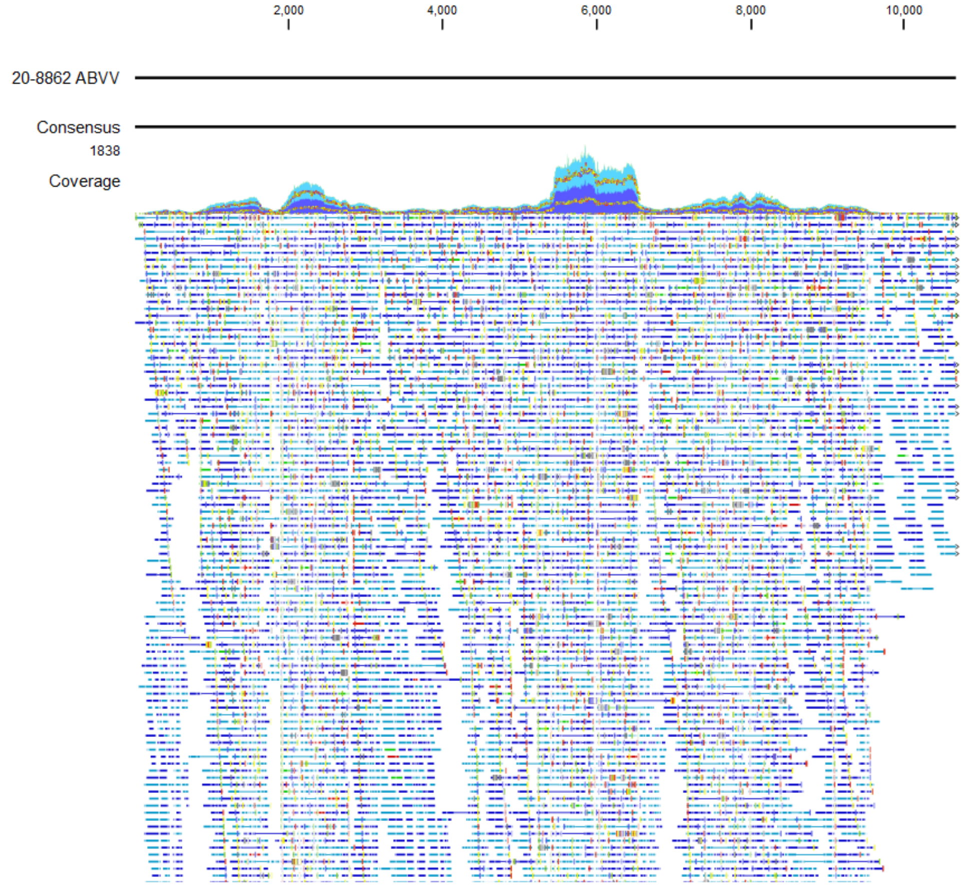
2,000
4,000
6,000
8,000
10,000
20-8862 ABVV
Consensus
1838
Coverage

d

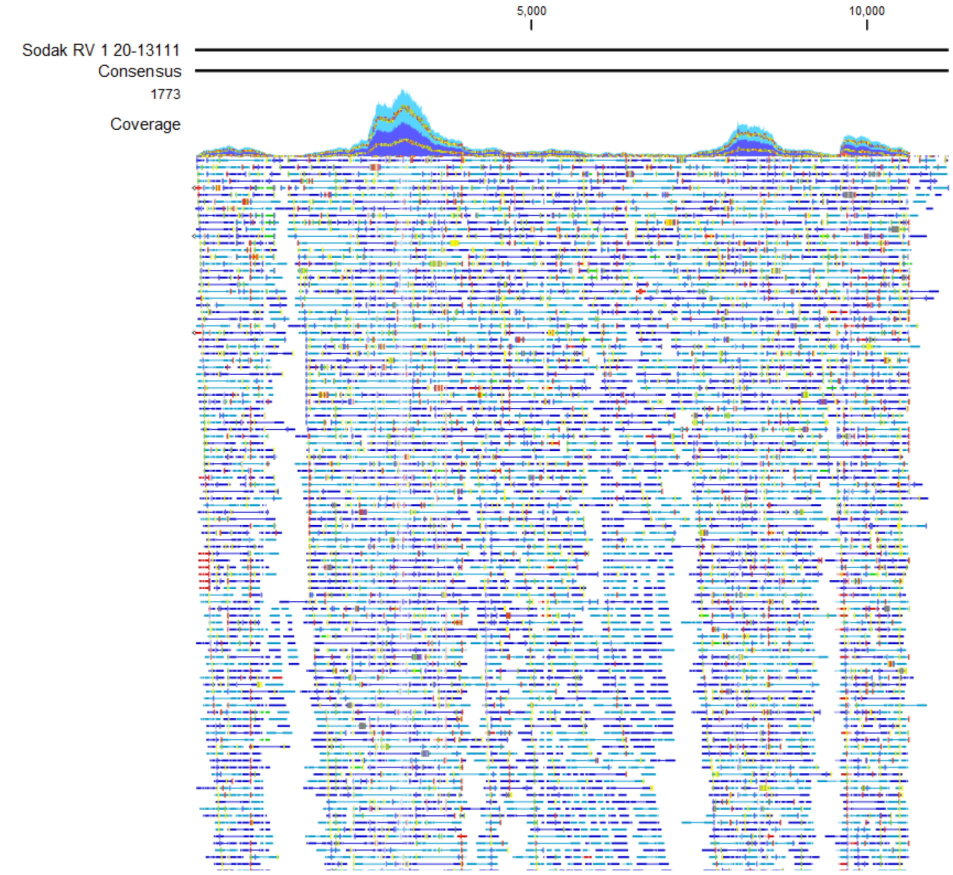

e

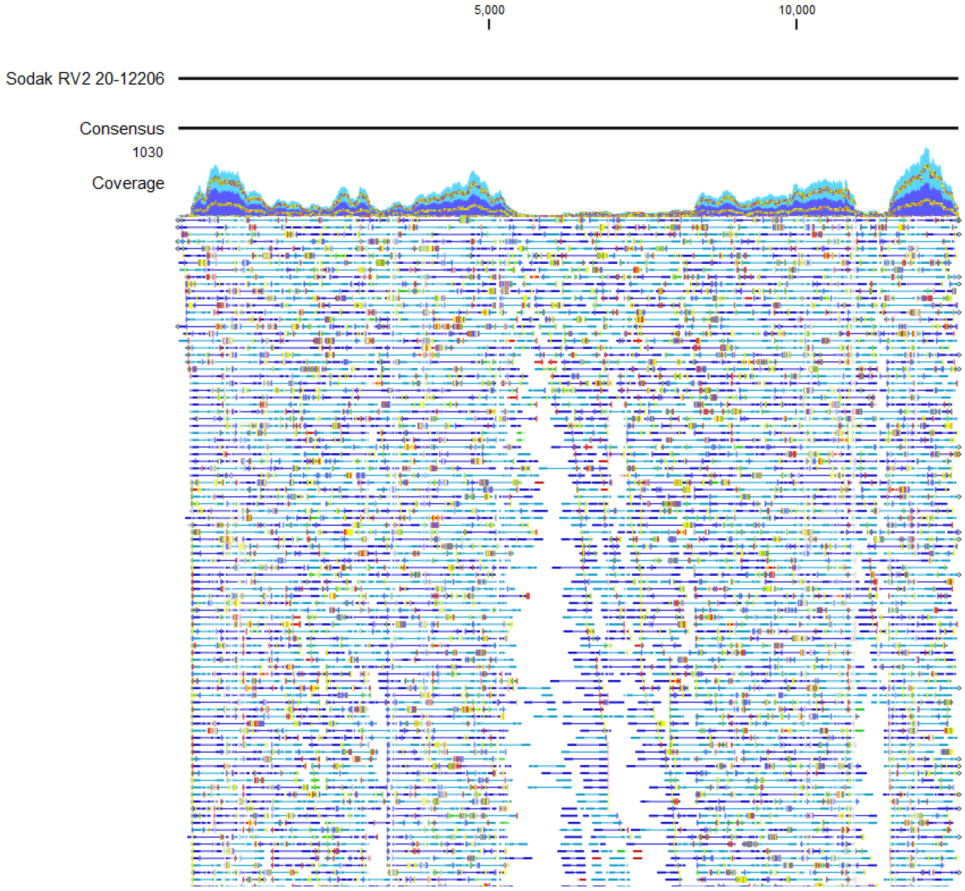

f

Figure S1. Sequencing reads mapping to Rhabdovirus genomes assembled de novo.

Table S1. Next generation sequencing metrics for samples containing rhabdoviruses.

	Total Reads	de novo assembled contig length (nt)	reads mapping to contig	average coverage	Ct
ABVV 20-0654	290,470	10,613	3,131	41.4x	21.2
ABVV 20-7011	337,220	10,719	289,416	3,660x	11.2
ABVV 20-7177	215,026	10,667	5,667	73.3x	18.5
ABVV 20-8862	504,160	10,614	51,303	533x	17.5
Sodak rhabdovirus 1	962,582	11,221	33,637	398.4x	24
Sodak rhabdovirus 2	568,376	12,600	25,391	296.9x	25.1

Table S2. BLASTP analysis of putative open reading frames. The percent identity is shown in each box along with protein length in parentheses (amino acids). NS, no similarity to known proteins by BLASTP analysis.

	N	P	M	G	L	Best BLASTP Hit
ABVV 20-0654	97.6 (424)	79.7 (232)	87.6 (211)	74.3 (506)	90.6 (2098)	ABVV (N,P,M,G,L)
ABVV 20-7011	97.6 (424)	80.2 (232)	87.6 (211)	74.3 (506)	90.9 (2096)	ABVV (N,P,M,G,L)
ABVV 20-7177	97.6 (424)	80.6 (232)	87.6 (210)	74.7 (506)	90.9 (2096)	ABVV (N,P,M,G,L)
ABVV 20-8862	97.6 (424)	80.2 (232)	87.6 (211)	74.3 (506)	90.0 (2098)	ABVV (N,P,M,G,L)
Sodak rhabdovirus 1	47.6 (427)	25.3 (304)	29.4 (214)	24.8 (519)	53.6 (2143)	Xingshan nematode virus 4 (N,P,M,G,L)
Sodak rhabdovirus 2	35.9 (471)	NS (378)	NS (213)	22.9 (517)	42.3 (2129)	Fujian dimarhabdovirus (N, L); Gata Virus (G)

Table S3. Nucleotide pairwise identity for American bat vesiculovirus (ABVV) strain genome sequences.

		1	2	3	4	5
ABVV 20-0654	1		99.06	99.23	99.23	76.78
ABVV 20-7177	2	99.06		99.23	99.24	76.80
ABVV 20-7011	3	99.23	99.23		99.40	76.84
ABVV 20-8862	4	99.23	99.24	99.40		76.77
ABVV TFFN-2013	5	76.78	76.80	76.84	76.77	

© 2020 by the authors. Submitted for possible open access publication under the terms and conditions of the Creative Commons Attribution (CC BY) license (http://creativecommons.org/licenses/by/4.0/).
